# Supplementary material for: Using a bistable animal opsin for switchable and scalable optogenetic inhibition of neurons
Source: EMBO Rep. 2021 Mar 2;22(5):e51866. doi: 10.15252/embr.202051866 (PMC8097317; doi:10.15252/embr.202051866)
Supplement: Supplementary file 1 — Expanded View Figures PDF [file EMBR-22-e51866-s001.pdf]

Expanded View Figures

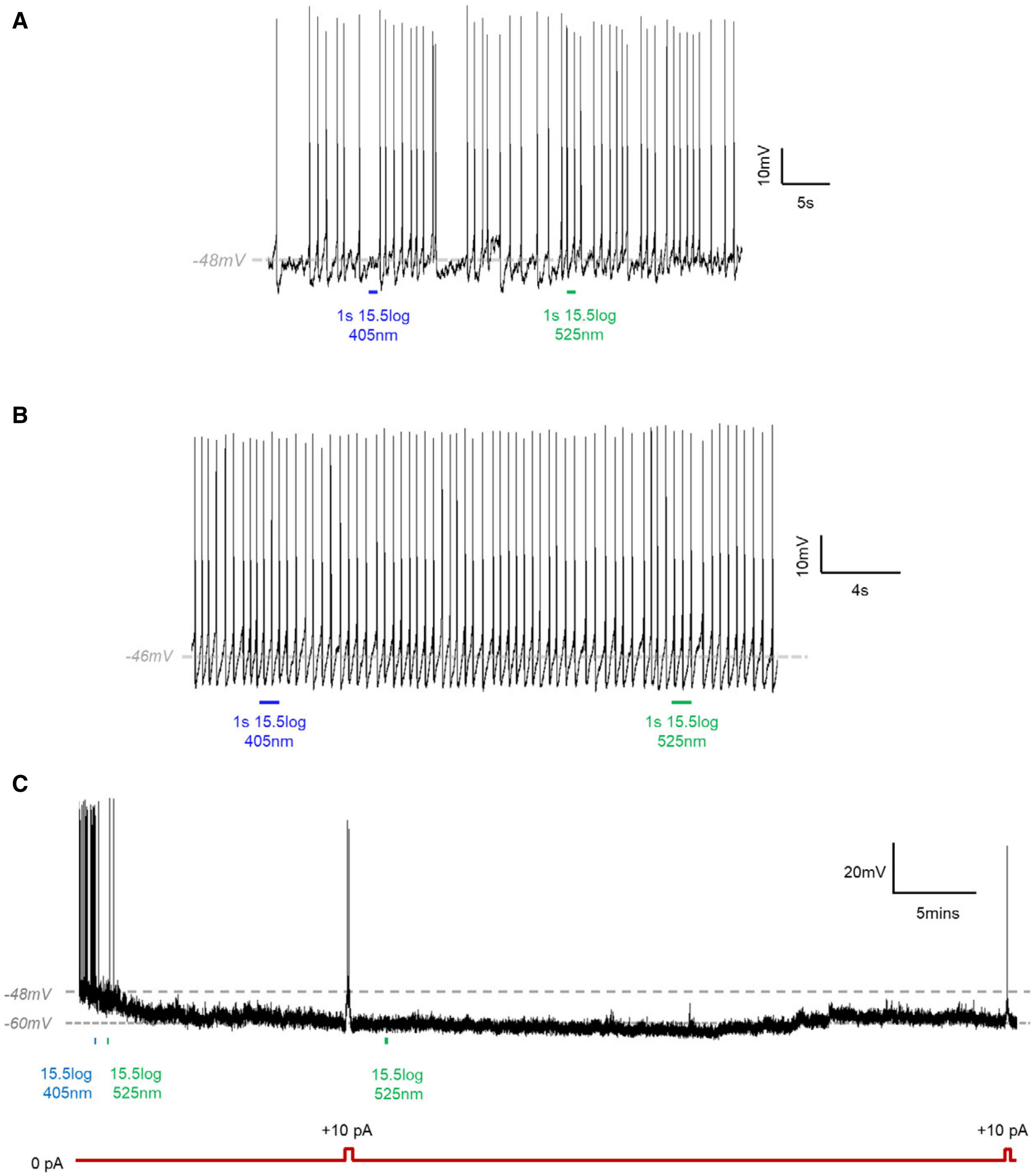

 © 2021 The Authors

Figure EV1. No responses to 405 nm light found in non-fluorescent SCN neurons and an example of hyperpolarised SCN neuron that did not respond to 525 nm light.

A, B Representative traces from 2 non-fluorescent cells in SCN exposed to 405 nm (1 s, blue) and 525 nm light (1 s, green). Neither cell shows hyperpolarisation or a change in firing rate in response to 405 nm light stimulus.

C Example trace from a cell with prolonged inhibition. After exposure to bright 405 nm light (blue), this cell remained hyperpolarised for at least 55 min with no obvious impact of 525 nm light (green). Hyperpolarisation was not due to decline in cell health, as action potentials could still be elicited when we manually applied a depolarising current. Schematic of depolarising pulse shown in red. We are not able to determine whether the failure of 525 nm light to restore firing reflects the Lamplight response or an unrelated change in spontaneous activity, as SCN neurons are known to switch between active and inactive states.

Data information: All light stimuli are 15.5log photons/cm^2/s. Timing of light stimuli shown by shaded vertical bars.

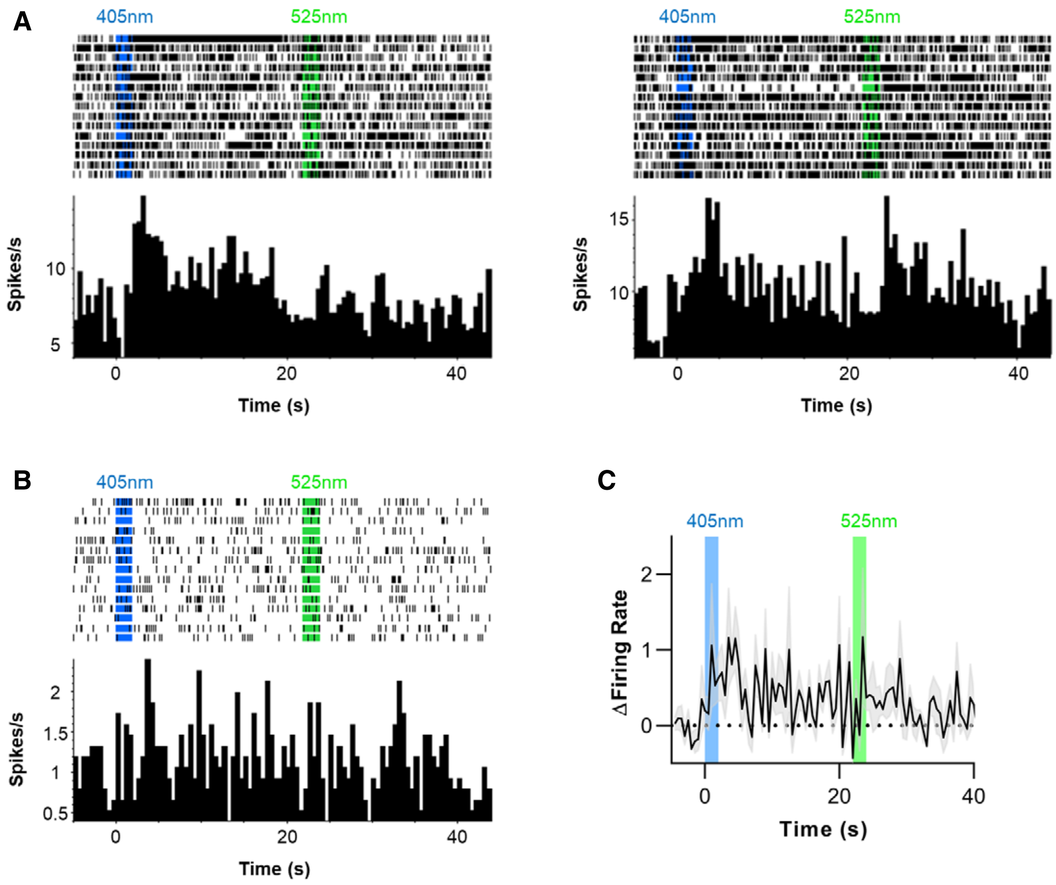

Figure EV2. Responses to 405 and 525 nm light in Lamplight-expressing retinas were absent in uninjected control.

A, B MEA recordings from uninjected *rd1* retina in response to 2 s 405 and 525 nm light. The objective statistical criteria used to identify putative light responsive single units applied to Lamplight retinas (Fig 3) returned 6 units classified as showing increases in firing following 405 nm light. Of these, 2 exhibited a slow and sustained excitation (especially to first stimulus) characteristic of ipRGC responses (A); the remainder did not show a repeatable light response across multiple stimulus presentations, indicating that they were likely false positives (representative in B).

C Mean firing rate for all putative "light responsive" units (N = 6) from uninjected retina. Error bars show SEM.

Data information: All representative retinal single units show perievent rasters (first trial at top) and associated perievent firing rate histograms (Bin size = 500 ms). Timing of light stimuli shown by shaded vertical bars. All light stimuli are 16 log total Lamplight effective photons/cm^2/s.

© 2021 The Authors
