# Supplementary material for: Using a bistable animal opsin for switchable and scalable optogenetic inhibition of neurons
Source: EMBO Rep. 2021 Mar 2;22(5):e51866. doi: 10.15252/embr.202051866 (PMC8097317; doi:10.15252/embr.202051866)
Supplement: Supplementary file 2 — Table EV1 [file EMBR-22-e51866-s003.docx]

***Table 1.*** **Ratiometric light stimuli used in Hek293T and retinal MEA experiments**

| **Light Stimuli** | **Lamplight Inactive state (λ_max_ = 370nm)**  **Effective Photons/cm^2^/s**  **(log photons)** | **Lamplight Active state (λ_max_ = 515nm)**  **Effective Photons/cm^2^/s**  **(log photons)** | **Total Lamplight**  **Effective Photons/cm^2^/s**  **(log photons)** | |
| --- | --- | --- | --- | --- |
| ***Hek293T BRET Assay*** | | | |  |
| **405nm only**  *Fig 1D, E*  *Fig 1F* | 1.93 x 10^15^ (15.29log)  4.83 x 10^14^ (14.68log) | 1.24 x 10^15^ (15.09log)  3.30 x 10^14^ (14.52log) | 3.15 x 10^15^ (15.50log)  8.12 x 10^14^ (14.91log) | |
| **525nm only**  *Fig 1D, E*  *Fig 1E*  *Fig 1F* | 1.50 x 10^12^ (12.18log)  6.43 x 10^11^ (11.81log)  1.13 x 10^12^ (12.05log) | 3.16 x 10^15^ (15.50log)  1.58 x 10^15^ (15.20log)  2.37 x 10^15^ (15.37log) | 3.20 x 10^15^ (15.50log)  1.58 x 10^15^ (15.20log)  2.37 x 10^15^ (15.37log) | |
| **Ratiometric stimuli (405nm : 525nm)**  *Fig 1H, 1I, 1J* | | | | |
| 1 : 0 | 3.19 x 10^15^ (15.50log) | 2.11 x 10^15^ (15.32log) | 5.30 x 10^15^ (15.72log) | |
| 0.8 : 0.2 | 2.58 x 10^15^ (15.41log) | 2.76 x 10^15^ (15.44log) | 5.34 x 10^15^ (15.73log) | |
| 0.6 : 0.4 | 1.93 x 10^15^ (15.28log) | 3.32 x 10^15^ (15.52log) | 5.24 x 10^15^ (15.72log) | |
| 0.4 : 0.6 | 1.28 x 10^15^ (15.11log) | 3.97 x 10^15^ (15.60log) | 5.25 x 10^15^ (15.72log) | |
| 0.2 : 0.8 | 6.71 x 10^14^ (14.83log) | 4.59 x 10^15^ (15.66log) | 5.26 x 10^15^ (15.72log) | |
| 0 : 1 | 7.21 x 10^12^ (12.86 log) | 5.19 x 10^15^ (15.72log) | 5.20 x 10^15^ (15.72log) | |
| ***Retinal MEA recordings*** | | | | |
| **405nm only**  *Fig 3B-3F* | 6.13 x 10^15^ (15.78log) | 4.08 x 10^15^ (15.61log) | 1.02 x 10^16^ (16.01log) | |
| **525nm only**  *Fig 3B-3F* | 3.96 x 10^12^ (12.59log) | 1.02 x 10^16^ (16.01log) | 1.02 x 10^16^ (16.01log) | |
| **Ratiometric stimuli (405nm: 525nm)** | | | | |
| *Fig 3E, 3F* | 3.06 x 10^15^ (15.49log) | 7.10 x 10^15^ (15.85log) | 1.02 x 10^16^ (16.01log) | |
|  |  |  |  | |
